# Selenomethionine (Se-Met) Induces the Cystine/Glutamate Exchanger SLC7A11 in Cultured Human Retinal Pigment Epithelial (RPE) Cells: Implications for Antioxidant Therapy in Aging Retina

**DOI:** 10.3390/antiox10010009

**Published:** 2020-12-24

**Authors:** Sudha Ananth, Seiji Miyauchi, Muthusamy Thangaraju, Ravirajsinh N. Jadeja, Manuela Bartoli, Vadivel Ganapathy, Pamela M. Martin

**Affiliations:** 1 Department of Biochemistry and Molecular Biology, Augusta University, Augusta, GA 30912, USA; SANANTH@augusta.edu (S.A.); seiji.miyauchi@phar.toho-u.ac.jp (S.M.); mthangaraju@augusta.edu (M.T.); rjadeja@augusta.edu (R.N.J.); 2 Culver Vision Discovery Institute, Augusta University, Augusta, GA 30912, USA; mbartoli@augusta.edu; 3 Department of Ophthalmology, Augusta University, Augusta, GA 30912, USA; 4 Department of Cell Biology and Biochemistry, Texas Tech Health Science Center, Lubbock, TX 79430, USA; vadivel.ganapathy@ttuhsc.edu; 5 Georgia Cancer Center, Augusta University, Augusta, GA 30912, USA

**Keywords:** retina, retinal pigment epithelium (RPE), oxidative stress, age-related macular degeneration (AMD), antioxidants, selenium, selenomethionine (Se-Met), SLC7A11, xCT, system xc, glutathione

## Abstract

Oxidative damage has been identified as a major causative factor in degenerative diseases of the retina; retinal pigment epithelial (RPE) cells are at high risk. Hence, identifying novel strategies for increasing the antioxidant capacity of RPE cells, the purpose of this study, is important. Specifically, we evaluated the influence of selenium in the form of selenomethionine (Se-Met) in cultured RPE cells on system xc- expression and functional activity and on cellular levels of glutathione, a major cellular antioxidant. ARPE-19 and mouse RPE cells were cultured with and without selenomethionine (Se-Met), the principal form of selenium in the diet. Promoter activity assay, uptake assay, RT-PCR, northern and western blots, and immunofluorescence were used to analyze the expression of xc-, Nrf2, and its target genes. Se-Met activated Nrf2 and induced the expression and function of xc- in RPE. Other target genes of Nrf2 were also induced. System xc- consists of two subunits, and Se-Met induced the subunit responsible for transport activity (SLC7A11). Selenocysteine also induced xc- but with less potency. The effect of Se-met on xc- was associated with an increase in maximal velocity and an increase in substrate affinity. Se-Met increased the cellular levels of glutathione in the control, an oxidatively stressed RPE. The Se-Met effect was selective; under identical conditions, taurine transport was not affected and Na+-coupled glutamate transport was inhibited. This study demonstrates that Se-Met enhances the antioxidant capacity of RPE by inducing the transporter xc- with a consequent increase in glutathione.

## 1. Introduction

Age-related macular degeneration (AMD) is the leading cause of blindness among persons age 60 and above in the U.S. and other highly developed nations. The repertoire of clinical and experimental studies implicating oxidative stress as a principal causative factor and retinal pigment epithelium (RPE) as a primary site of pathology in the disease is quite large and steadily expanding [1,2,3,4]. RPE cells generate a substantial amount of reactive oxygen species (ROS) as a consequence of their normal physiologic functions (i.e., light absorption/photo-oxidation, phagocytosis of lipid-rich outer segments, etc.) [5]. Interestingly, they are at the same time equipped with cellular machinery to counter the potentially detrimental effects of ROS. The risk of oxidative damage to these cells is therefore influenced critically by the ratio of oxidant generation to the corresponding activity of endogenous cellular antioxidant defenses. Interestingly, increased age, the major risk factor for AMD, is associated with a progressive and irreversible accumulation of ROS in most major cell/tissue types, including RPE/retina, and a diminished capacity for protection against oxidative stress. This is compounded further by genetic and environmental factors that contribute to disruption of the delicate balance between pro- and antioxidant factors in favor of pro-oxidants/increased oxidative stress [6,7]. Given the high susceptibility of RPE to oxidant-induced damage, identification of novel strategies for protecting these cells and potentially, in turn, slowing the development/progression of this sight-debilitating disease is critically important.

Glutathione (GSH) is the major antioxidant responsible for the protection of RPE against oxidant-induced damage/dysfunction [8]. The ability of cells to synthesize GSH depends largely upon the availability of cysteine, a factor that hinges critically upon the activity of the cystine-glutamate exchanger, also known as system xc- [9,10]. System xc- mediates the Na+-independent entry of cystine into cells coupled to the efflux of intracellular glutamate. Extracellularly, glutamate participates in neurotransmission and other signaling pathways, whereas cystine is rapidly reduced intracellularly to cysteine, the rate-limiting factor in GSH synthesis. Aside from those functions, the transport system has also been shown to play an essential role in the regulation of cell cycle progression [11]. System xc- is therefore a critical regulator of endogenous GSH synthesis, cellular redox balance, and ultimately cell fate [12,13]. As such, strategies to induce the expression and function of this transport system have a direct impact not only on the normal biology of RPE cells but also on the ability of these and other retinal cells to respond effectively to oxidative stress [12,14]. Thus, the potential therapeutic value of such strategies is high.

In the present study, we evaluated selenomethionine (Se-Met), an organic form of selenium, for this very purpose. Specifically, we show that, in cultured human retinal pigment epithelial cells, selenium in the form of Se-Met induces nuclear factor erythroid 2-related factor 2 (Nrf2) expression; upregulates the expression of xCT, the functional subunit of the system xc- transport system; and therefore positively influences the production of GSH. Previous studies have demonstrated the natural antioxidant properties of selenium-containing compounds, a property thought to be related primarily to their incorporation into selenoproteins like the glutathione peroxidases (GPXs) and thioredoxin reductases (TXNRDs) [15,16]; however, our present findings provide a novel additional mechanism to explain the antioxidant feature of these compounds.

## 2. Materials and Methods

### 2.1. Materials

Reagents were obtained from the following sources: unlabeled amino acids (Sigma-Aldrich; St. Louis, MO, USA); TRIzol and Lipofectamine 2000 (Invitrogen-Gibco; Carlsbad, CA, USA); GeneAmp RT-PCR kit (Applied Biosystems/Life Technologies; Carlsbad, CA, USA); Taq polymerase kit (TaKaRa; Mountain View, CA, USA); Power Block (Biogenex; San Ramon, CA, USA); GSH-Glo Glutathione Assay Kit, pUIIR3-EGFP and pGL3-Basic vectors (Promega; Madison, WI, USA); rabbit polyclonal anti-human Nrf2 antibody (AbCam; Cambridge, MA, USA); Fluoromount mounting media (Sigma, St. Louis, MO, USA); Enhanced ChemiLuminescence kit (PerkinElmer Life Sciences, Waltham, MA, USA); Bio-Rad RC DC protein assay kit (Bio-Rad; Hercules, CA, USA); Pierce Pinpoint Cell Surface Isolation kit (Thermoscientific; Rockford, IL, USA); and [3H]-glutamate and [3H]-taurine (Perkin Elmer Life Sciences; Boston, MA, USA). Rabbit polyclonal anti-xCT antibody was a generous gift from Dr. Sylvia B. Smith [17]. Culture media and supplements, goat anti-rabbit Alexa Fluor 568 secondary antibody, and Hoechst 33342 stain were from Invitrogen-Gibco (Carlsbad, CA, USA).

### 2.2. Animals and Cell Culture

Normal C57BL/6J mice (males, aged 3 weeks) were obtained from the Jackson Laboratory (Bar Harbor, ME) and used to establish primary cultures of RPE per our previously published method [18,19,20]. All animals used in the study were maintained in accordance with the ARVO Statement for the Use of Animals in Ophthalmic and Vision Research and were approved by the Institutional Committee for Animal Use in Research and Education of the Augusta University (2009–0214). Human RPE cells (ARPE-19) were obtained from American Type Culture Collection (Manassas, VA, USA). Cell viability was evaluated using trypan blue exclusion assay. All experiments, other than immunofluorescence studies in which it was necessary to evaluate single cells (see below in Section 2.6. “Immunofluorescence Analysis”), were performed using completely confluent, highly differentiated ARPE-19 cultures, and the key findings were confirmed in primary mouse RPE.

### 2.3. RT (Reverse Transcription)-PCR

RNA was isolated from ARPE-19 cells cultured in the presence or absence of Se-Met (1 mM, 16 h) and used for RT-PCR using the GeneAmp RT-PCR kit. System xc- is a heterodimeric protein made up of xCT, the transport subunit, and 4F2hc, the subunit responsible for trafficking of xCT to the plasma membrane. We analyzed the expressions of both xCT and 4F2hc. Primer sequences and expected product sizes for xCT and 4F2hc have been described [21]. Information specific to the other primers used in the study is provided in Supplementary Table S1. In all PCR reactions, 18 S ribosomal RNA served as the internal control. The molecular identity of the PCR products was confirmed by sequencing analysis.

### 2.4. Northern Blot Analysis

Northern blotting was performed to analyze the steady-state levels of xCT mRNA and 4F2hc mRNA in ARPE-19 cells cultured in the presence or absence of selenomethionine (1 mM, 16 h) following the method of Bridges et al. [10]. β-Actin mRNA was monitored as an internal control.

### 2.5. Luciferase Promoter Assay

SLC7A11, the gene coding for human xCT, maps to chromosome 4, at 4q28-q32. The promoter region of this gene contains a putative ARE-Nrf2 binding site (Genbank accession number NT_016354) [22]. To evaluate whether the effects of Se-Met on xCT expression are mediated via transcription, luciferase promoter assay was performed following our previously published method [23]. Two different SLC7A11-specific promoter constructs were designed; the first, which we dubbed SLC7A11 promoter-long, was designed such that the portion of the promoter region amplified included the purported ARE-Nrf2 binding site and was approximately 780 bp in length. The second, SLC7A11 promoter-short, was designed such that the region selected for amplification was located just downstream of the ARE-Nrf2 binding site, and in turn, was only approximately 400 bp in length. SLC7A11 promoter-long- and SLC7A11 promoter-short-luciferase constructs were generated by first subcloning the 0.7 and 0.4-kb promoter fragments (obtained by PCR using human genomic DNA as the template), respectively, into the pGEM-T Easy vector and then by cloning the XhoI/NcoI-digested promoter inserts into the pGL3-Basic luciferase vector. The primers used for PCR were 5′-AACTCGAGGACTGTATTGCCTT-3′ (forward, promoter-long), 5′-AACTCGAGGCTGGAGGCTTCTC-3′ (forward, promoter-short), and 5′-TGACCATGGTCATAGTAGGGACA-3′ (reverse, NcoI site/pGL3-Basic). All constructs were verified by sequencing. ARPE-19 cells (approximately 80% confluent) were transfected with the respective plasmids using Lipofectamine 2000 according to the manufacturer’s instructions (Invitrogen). Se-Met (1 mM) was added to the culture medium of “treated” cells 32 h post-transfection. Cells were collected, and lysates were prepared 48 h post-transfection. Luciferase activity was measured using a Tecan microplate reader (Tecan US, Inc., Morrisville, NC, USA) and was used as an indicator of promoter activation. Luciferase activity was normalized for protein levels and compared with empty vector-transfected control cells.

### 2.6. Immunofluorescence Analysis

ARPE-19 cells were seeded on glass coverslips and cultured in the presence or absence of Se-Met (1 mM) for 16 h. Cells were then air-dried, fixed in 4% (*w/v*) paraformaldehyde, washed with 0.01 M PBS (pH 7.4), and blocked with 1× Power Block for 1 h at room temperature. Following overnight incubation at 4 °C with anti-xCT (1:500), anti-Nrf2 antibody (1:100), or PBS (negative controls), cells were again washed in PBS and incubated for 45 min at room temperature with goat anti-rabbit Alexa Fluor 568 secondary antibody at a dilution of 1:2000. Following a final PBS wash, cells were incubated at room temperature with Hoechst 33342 (1:10,000), a nuclear counterstain, and coverslips were mounted on standard glass microscope slides using Fluoromount mounting media. Epifluorescence visualization of immunolabeled proteins was done using the Zeiss Axioplan-2 microscope, Axiovision software, and an HRM camera.

### 2.7. Western Blot Analysis

Western blotting was performed to detect Nrf2 or xCT in protein lysates prepared from ARPE-19 cells cultured in the presence or absence of Se-Met (1 mM, 16 h). For analysis of Nrf2 expression, protein lysates were subjected to SDS-PAGE, then transferred to nitrocellulose membranes, and incubated with antibody against Nrf2 overnight at 4 °C. Membranes were then incubated with horseradish peroxidase-conjugated anti-rabbit IgG antibody and washed, and antibody-specific bands were detected using chemiluminescence. xCT protein localizes both intracellularly and on the plasma membrane; however, it is the protein present on the plasma membrane that is functionally active, responsible directly for the transport activity. As such, to evaluate the effects of Se-Met treatment on the plasma membrane versus cytosolic fraction of xCT protein, membrane proteins were biotinylated using the Pinpoint Cell Surface Isolation Kit according to the manufacturer’s instructions and the method of Mysona et al. [24]. In brief, cells were labeled with thiol-cleavable amine-reactive biotinylation reagent and then lysed in the presence of a mild detergent. Labeled membrane proteins were collected using a column containing immobilized NeutrAvidin gel; the intracellular protein fraction was present in the eluate. Membrane proteins were released by treatment of the column with SDS-polyacrylaminde gel sample buffer containing 50 mM DTT, protein concentrations were determined via Bio-Rad RC DC protein assay, and western blotting was performed. β-Actin served as the loading control.

### 2.8. Assay of System xc- Transport Activity

System xc- is a Na+-independent transport system that, under physiologic conditions, mediates cellular entry of cystine in exchange for intracellular glutamate. However, the activity of this transporter is routinely measured by monitoring the cellular uptake of [3H]-glutamate rather than [3H]-cystine under Na+-free conditions, particularly in RPE5 [25]. This because, in addition to system xc-, RPE cells express a transport system known as b0,+ which can also contribute to the influx of cystine. However, when glutamate is used as the substrate, the uptake appears to be mediated exclusively by system xc-. Hence in the present study, the transport activity of system xc- was monitored as the influx of [3H]-glutamate in exchange for intracellular unlabeled glutamate. Specifically, we measured the uptake of [3H]-glutamate (25 μM) in ARPE-19 cells or primary mouse RPE cells cultured in the presence or absence of Se-Met, selenocysteine (Se-Cys), S-adenosyl methionine (SAM), glutamate (Glu), cystine (Cys), methionine (Met), cysteine (Cys), glutamine, phenylalanine, aspartic acid, arginine, lysine, valine, or alanine at 1 or 5 mM using a Na+-free uptake buffer [21]. Kinetic analysis of system xc- activity in cells cultured in the presence or absence of Se-Met was performed by measuring the transport activity over a wide range of glutamate concentrations (2.5–1000 μM) and by analyzing the data according to the Michaelis–Menten equation describing a single saturable transport system. Kt and Vmax were calculated using the nonlinear regression method and then confirmed using the linear regression method. xc- is not the sole transport system responsible for mediating glutamate uptake in mammalian cells; members of the excitatory amino acid transporter (EAAT) family also contribute to the uptake. To determine whether the activity of EAATs (Na+-dependent glutamate transporters) is also influenced by Se-Met, uptake of [3H]-glutamate was also monitored in a Na+-containing buffer (25 mM Hepes/Tris (pH 7.5), 140 mM NaCl, 5.4 mM KCl, 1.8 mM CaCl2, 0.8 mM MgSO4, and 5 mM glucose).

### 2.9. Hydrogen Peroxide (H_2_O_2_) Treatment and Glutathione Measurement

ARPE-19 cells were seeded in 6-well plates (0.2 × 106 cells/well) and grown to approximately 80% confluence. Cells were pretreated for 1 h with or without Se-Met (1 mM), followed by 2 h exposure to 500 μM H_2_O_2_. The concentration of H_2_O_2_ used in these studies was determined based upon prior experimentation [21]. Cellular glutathione levels were measured using the GSH Glo assay kit as previously described [21].

### 2.10. Data Analysis

All cell treatments were performed at least in triplicate. All data are expressed as mean ± S.E.M. Student’s *t*-test (normally distributed data) or the Mann–Whitney U test (nonparametric data) were used to determine significance. *p* < 0.05 was considered to be statistically significant.

## 3. Results

### 3.1. Se-Met-Induced Upregulation of Nrf2

**Figure 1:**
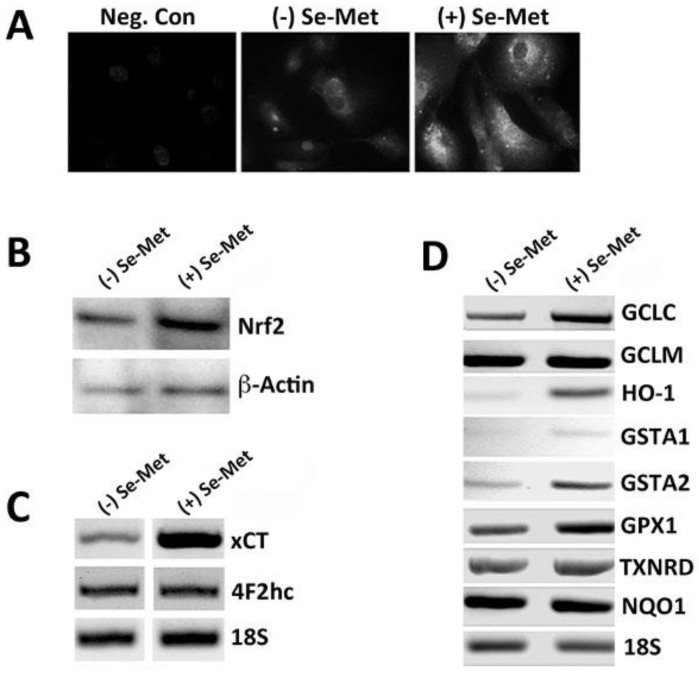
Selenomethionine (Se-Met)-induced upregulation of Nrf2 in ARPE-19 cells: (**A**) immunocytochemical analysis of Nrf2 protein in ARPE-19 cells cultured in the presence (+Se-Met) or absence (−Se-Met) of selenomethionine (1 mM, 16 h), where cells in which PBS was substituted for the primary antibody served as the negative control (Neg. Con); (**B**) western blot analysis of Nrf2 in control and Se-Met-treated ARPE-19 cells; (**C**) RT-PCR analysis of mRNA transcripts specific for xCT (SLC7A11) and 4F2hc in control (−Se-Met) and Se-Met-treated ARPE-19 cells; and (**D**) RT-PCR analysis of Nrf2 target genes: glutamate-cysteine ligase catalytic subunit (GCLC), glutamate-cysteine ligase regulatory subunit (GCLM), heme oxygenase-1 (HO-1), glutathione S-transferase A1 (GSTA1), glutathione S-transferase A2 (GSTA2), thioredoxin reductase (TXNRD), glutathione peroxidase (GPX), and NAD(P)H oxidase: quinone 1 (NQO1), in ARPE-19 cells cultured in the presence (+Se-Met) or absence (−Se-Met) of selenomethionine (1 mM, 16 h).

The antioxidant properties of selenium are well documented, particularly with respect to their usefulness in the treatment/prevention of cancer-related pathologies [26]. Such properties are thought to be a direct consequence of the induction of the Nrf2/phase II antioxidant response pathway. Interestingly, compounds capable of inducing Nrf2 and its target genes have been shown to be similarly protective of retinal cells, particularly RPE [27,28]; however, to our knowledge, whether selenium elicits a similar effect on Nrf2/phase II antioxidant responses in RPE has not been studied. Given our present interest in exploring selenium as a potential antioxidant therapy for preservation of RPE and prevention of retinal pathology, we exposed human RPE (ARPE-19) cells to Se-Met, the primary form of selenium in the diet and evaluated Nrf2 protein expression using immunofluorescence and western blotting. A positive signal (red fluorescence) indicative of Nrf2 protein expression was detectable in control, untreated cells (Figure 1A). The intensity of this signal was increased significantly in Se-Met-treated cells. To confirm these findings, additional cells were cultured for 4–6 weeks to facilitate proper differentiation. The protein was then isolated from these highly differentiated cells cultured and used for western blotting; a similar increase in Nrf2 expression was detected using this method (Figure 1B). Nrf2 induces several genes crucial to the cellular defense against oxidative stress, including xCT. As such, using RNA obtained from cells treated identically as described above, we evaluated the expression of xCT (SLC7A11) and 4F2hc, the catalytic and regulatory subunits of system xc-, respectively, by RT-PCR (Figure 1C). xCT expression increased robustly in Se-Met-treated cells. The expression of 4F2hc was not affected. We evaluated also the expression of a number of other well-known Nrf2 target genes and/or selenoproteins including glutamate-cysteine ligase catalytic subunit (GCLC), glutamate-cysteine ligase regulatory subunit (GCLM), heme oxygenase 1 (HO-1), glutathione S-transferase A1 (GSTA1), glutathione S-transferase A2 (GSTA2), glutathione peroxidase 1 (GPX1), thioredoxin reductase (TXNRD), and NAD(P)H oxidase/quinone 1 (NQO1) (Figure 1D). Indeed, we found the expression of a number of these genes to be upregulated also in Se-Met-treated cells.

### 3.2. Se-Met-Induced Upregulation of xCT Expression

**Figure 2:**
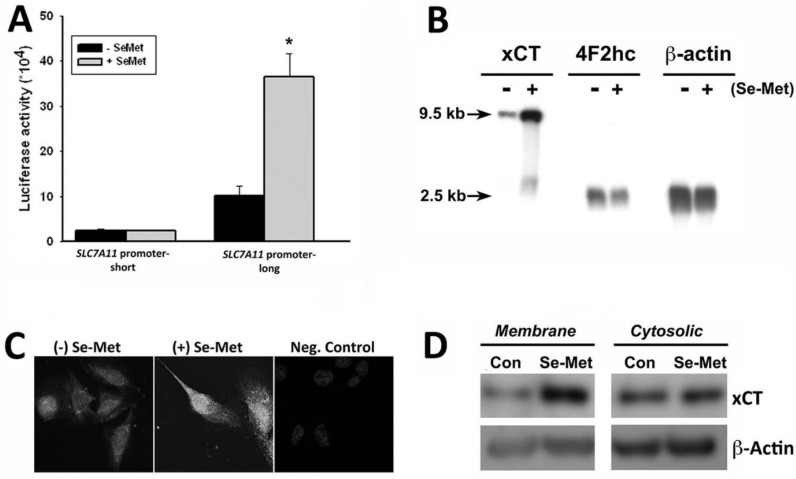
Se-Met-induced expression of xCT (SLC7A11): (**A**) luciferase reporter assay of Se-Met (1 mM)-induced SLC7A11 (xCT) promoter activation in in ARPE-19 cells transfected with luciferase reporter constructs containing either the short or long form of SLC7A11 promoter (* *p* < 0.001 compared to the control (−Se-Met) SLC7A11 promoter-long cells); (**B**) northern blot analysis of xCT and 4F2hc expression in control and Se-Met-treated ARPE-19 cells; (**C**) immunocytochemical analysis of xCT protein in ARPE-19 cells cultured in the presence (+Se-Met) or absence (−Se-Met) of selenomethionine (1 mM, 16 h), where cells in which PBS was substituted for primary antibody served as the negative control (Neg. Con); and (**D**) western blot analysis of xCT protein in plasma membrane versus cytosolic protein fractions isolated from ARPE-19 cells cultured in the presence or absence of Se-Met (1 mM, 16 h).

Given our specific interest in mechanisms capable of stimulating system xc- and, in turn, endogenous GSH synthesis, we sought to explore this phenomenon further. As stated above, xCT is coded for by SLC7A11, an Nrf2 target gene [29]. Though we show increased expressions of both Nrf2 and xCT in conjunction with Se-Met treatment of ARPE-19 cells, we have not established the cause-and-effect relationship between the two. Under unstressed conditions, Nrf2 resides in the cytoplasm, complexed to Kelch-like ECH-associated protein (Keap1); this is its inactive state. This complex is disrupted by oxidative stress, and Nrf2 is released and translocated into the nucleus. Nrf2 then binds antioxidant response elements (ARE) in the promoters of its target genes, thereby inducing their expression. The presence of an ARE binding site within the promoter region of SLC7A11 (xCT) has been reported [29,30]. To determine whether the Se-Met-induced upregulation of xCT observed in our experimental system is due to Nrf2-dependent induction of the SLC7A11 promoter activity, we designed two SLC7A11-specific promoter constructs: one containing the ARE-binding site (SLC7A11 promoter-long) and another in which the ARE-site was excluded (SLC7A11 promoter-short), each driving an EGFP or luciferase reporter. Luciferase activity in vector control cells was negligible irrespective of Se-Met exposure (Figure 2A) but was increased significantly in ARPE-19 cells transfected with SLC7A11 promoter-long and exposed to Se-Met (*p* < 0.001). These data strongly support the Se-Met-induced activation of ARE/Nrf2 in the promoter region of xCT (SLC7A11). The RT-PCR results above showing upregulated xCT expression upon Se-Met treatment (Figure 1C) are consistent with this prediction; however, we confirmed the RT-PCR data by northern blot (Figure 2B). To evaluate whether the Se-Met-induced increase in xCT mRNA is associated with a similar increase in xCT protein, ARPE-19 cells were cultured identically as described above and xCT protein was analyzed by immunofluorescence (Figure 2C) and western blotting (Figure 2D). Both methods revealed robust increases in xCT protein in the presence of Se-Met, and the increase was specifically found in the transporter density in the plasma membrane.

### 3.3. Characterization of Se-Met-Induced xc- Transport Activity

**Figure 3:**
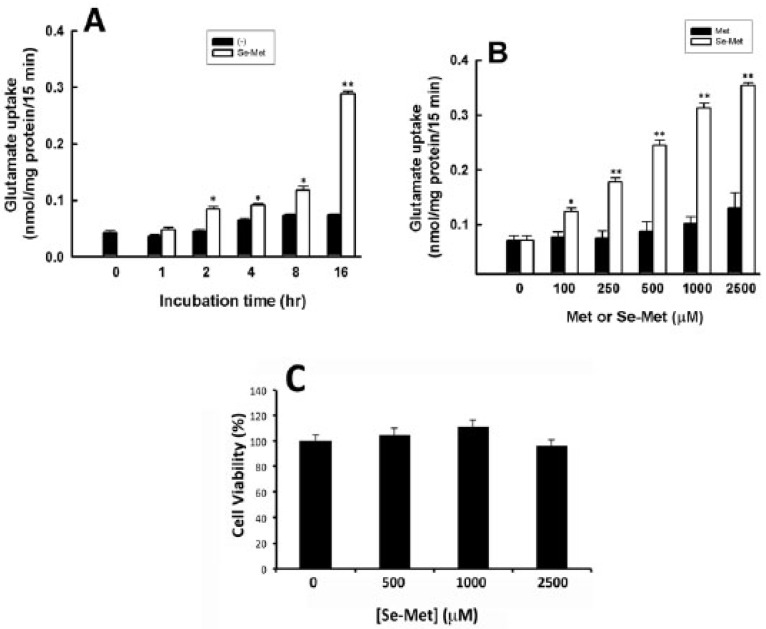
Selenomethionine (Se-Met)-induced stimulation of xc- transport activity is time- and dose-dependent. (**A**) Uptake of [3H]-glutamate (2.5 μM) was measured for 15 min in ARPE-19 cells cultured in the presence or absence of Se-Met (1 mM) for 0–16 h. Uptake was monitored at 37 °C in the absence of Na+. (**B**) Na+-independent [3H]-glutamate (2.5 μM) uptake was measured for 15 min in ARPE-19 cells cultured in the presence or absence of varying concentrations (0–2500 μM) of selenomethionine (Se-Met) or methionine (Met) for 16 h. (* *p* < 0.05; ** *p* < 0.01; compared to corresponding concentration of Met). (**C**) Cell viability was determined by trypan blue exclusion assay.

Confluent ARPE-19 cell cultures were exposed to in Se-Met (1 mM) for 0–16 h, followed by the monitoring of [3H]-glutamate uptake under Na+-free conditions, a well-established read-out of system xc- transport activity. With respect to time-dependency, the greatest stimulation in xc- transport activity was observed in cells exposed to Se-Met for 16 h (Figure 3A). The dose-dependency of the effect of Se-Met was also evaluated. Significant stimulation of xc- transport activity was observed in the presence of Se-Met at concentrations as low as 100 μM and increased steadily with increasing doses of Se-Met (Figure 3B). To confirm that the increase in system xc- transport activity observed in the presence of higher doses of Se-Met was not an artifact of cellular stress or toxicity, cell viability was confirmed via trypan blue exclusion assay (Figure 3C). Indeed, the viability of the cells was not affected by Se-Met at any of the concentrations used in the present study.

**Figure 4:**
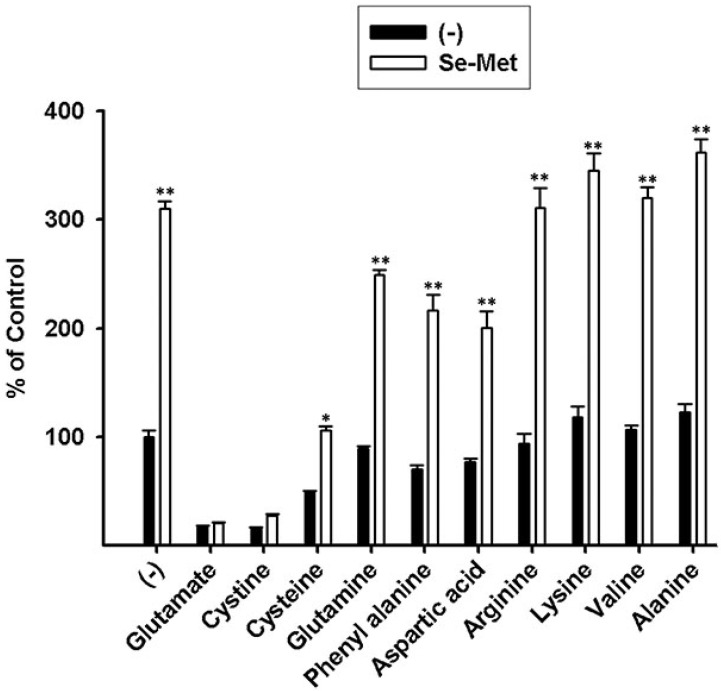
Substrate specificity of system xc- in control and selenomethionine (Se-Met)-treated ARPE-19 cells: uptake of [3H]-glutamate (2.5 μM) was measured in control and Se-Met-treated ARPE-19 cells in the absence of Na+ for 15 min at 37 °C and in the presence or absence of excess unlabeled amino acids, each at a concentration of 5 mM (* *p* < 0.01; ** *p* < 0.001).

**Figure 5:**
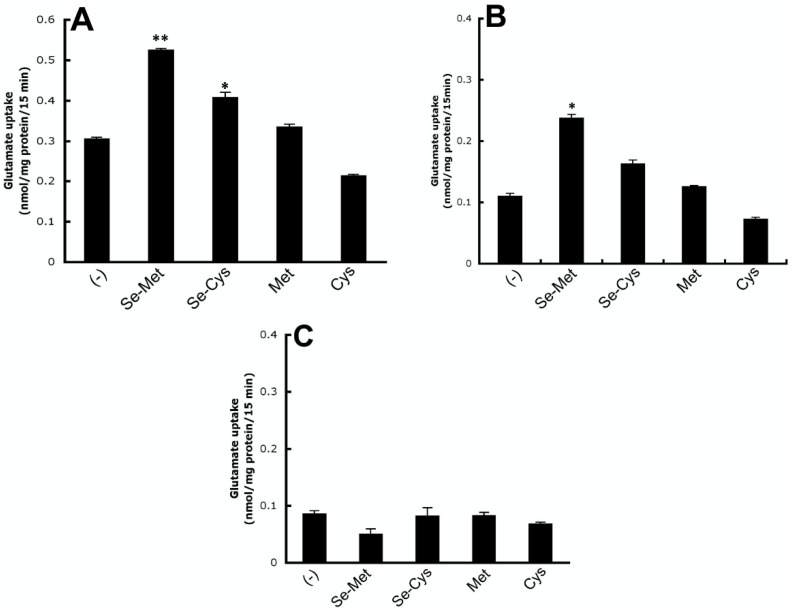
Differential influence of selenoamino acids (Se-Met and Se-Cys) and sulfur amino acids (Met and Cys) on system xc- transport activity: ARPE-19 cells were cultured in the presence or absence of selenomethionine (Se-Met), selenocysteine (Se-Cys), methionine (Met), and cysteine (Cys), each at 1 mM, for 16 h. Uptake of [3H]-glutamate (2.5 μM) was measured for 15 min at 37 °C in the presence and absence of Na+: (**A**) Na+-independent, xc- -specific glutamate uptake; (**B**) total glutamate uptake; and (**C**) Na+ -dependent, excitatory amino acid transporter (EAAT)-specific glutamate uptake. (* *p* < 0.05, ** *p* < 0.01 compared to control).

To confirm the contribution of system xc- to the observed Na+-dependent uptake of [3H]-glutamate, we evaluated the substrate selectivity of the uptake process. Uptake of [3H]-glutamate in the control and Se-Met-treated ARPE-19 cells was evaluated in the presence of various unlabeled amino acids at a concentration of 5 mM. The uptake of [3H]-glutamate was inhibited robustly by cystine and unlabeled glutamate. Inhibition by cysteine was much less. Other amino acids examined had no or little inhibitory effect (Figure 4). System xc- is selective for cystine and glutamate, and the observed substrate selectivity of the Se-Met-induced transport system responsible for [3H]-glutamate uptake in ARPE-19 cells confirms the identity of system xc-.Se-Met, though the major form of selenium present in the diet is not the sole form attainable from dietary sources; selenocysteine (Se-Cys) also represents a dietary source of selenium [31]. Selenoamino acids such as Se-Met and Se-Cys can be incorporated directly into selenoproteins such as TXNRDs and GPXs or alternately substituted for methionine (Met) or cysteine (Cys), respectively, in other biologic reactions. In fact, the random substitution of selenoamino acids for sulfur-containing amino acids is not thought to substantially alter protein structure or function [32]. As such, here, we evaluated the differential effects of Se-Met, Se-Cys, Cys, and Met on system xc- transport activity. ARPE-19 cells were cultured in the presence or absence of 1 mM Se-Met, Se-Cys, Cys, or Met (16 h incubation), and the Na+-independent uptake of [3H]-glutamate was monitored. As observed earlier (Figure 3 and Figure 4), xc- activity almost doubled in the presence of Se-Met (Figure 5A). Moderate stimulation was observed also in the presence of Se-Cys; however, Met and Cys had little to no effect on xc- transport activity.

Members of the excitatory amino acid transporter (EAAT) family also mediate glutamate uptake, but only in a Na+-dependent manner. Many members of the EAAT family are expressed in retina; RPE specifically expresses at least two subtypes: EAAT3 (EAAC1) and EAAT4 [33]. As such, in addition to evaluating the differential influence of Cys and Met versus Se-Met and Se-Cys on Na+- independent glutamate uptake/system xc- transport activity, we asked also whether these compounds influence the cellular uptake of glutamate in RPE accomplished by EAATs. ARPE-19 cells were cultured identically as described above, and the uptake of [3H]-glutamate was monitored in the presence of Na+ (Figure 5B). Under these conditions, glutamate uptake was again stimulated significantly in the presence of Se-Met and moderately by Se-Cys; Met and Cys had very little effect. It is important to note however that, when conducting uptake assays in the presence of Na+, both Na+-dependent and Na+-independent mechanisms of glutamate transport are at work; hence, glutamate uptake under these conditions is representative of the “total” glutamate uptake mediated by xc- and EAATs. The specific contribution of EAATs to glutamate uptake is determined by calculating Na+-coupled uptake (i.e., uptake in the presence of Na+ minus uptake in the absence of Na+). These calculations showed that the activity of EAATs was not influenced by Se-Met, Se-Cys, Met, or Cysteine (Figure 5C).

### 3.4. Kinetic Analysis of Se-Met-Induced System xc- Transport Activity

**Figure 6:**
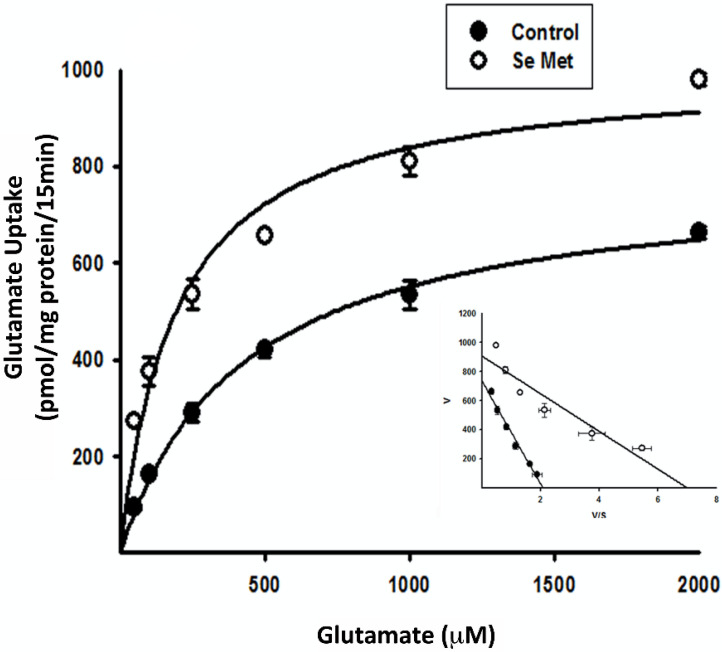
Kinetic analysis of system xc- activity in the presence or absence of Se-Met in ARPE-19 cells: saturation kinetics of glutamate uptake in ARPE-19 cells cultured for 16 h in the presence or absence of 1 mM selenomethionine (Se-Met) was evaluated by monitoring the uptake of [3H]-glutamate in Na+-free medium with various concentrations of unlabeled glutamate (0–2000 μM). Inset: Eadie–Hofstee plot.

To gain a better understanding of the effects of Se-Met on the transport system, we performed additional studies to determine which of the kinetic parameters of the system (xc-)-mediated transport process are affected by this compound (Figure 6). Both kinetic parameters, namely the Michaelis constant (Kt) and the maximal velocity (Vmax), were affected. Kt was reduced from 353 ± 24 μM to 129 ± 24 μM in the presence of Se-Met (1 mM) (*p* < 0.01); Vmax was increased from 0.74 ± 0.03 nmol/106 cells/min to 0.91 ± 0.07 nmol/106 cells/min (*p* < 0.05). Eadie–Hofstee transformation of the data is provided in the inset of Figure 6. These data demonstrate that the increase in activity of system xc- as a result of treatment of the cells with Se-Met is associated with an increase in maximal velocity (increase in Vmax) and an increase in substrate affinity (decrease in Kt).

### 3.5. Influence of Se-Met on Glutathione Levels in ARPE-19 Cells with and without Oxidative Stress

**Figure 7:**
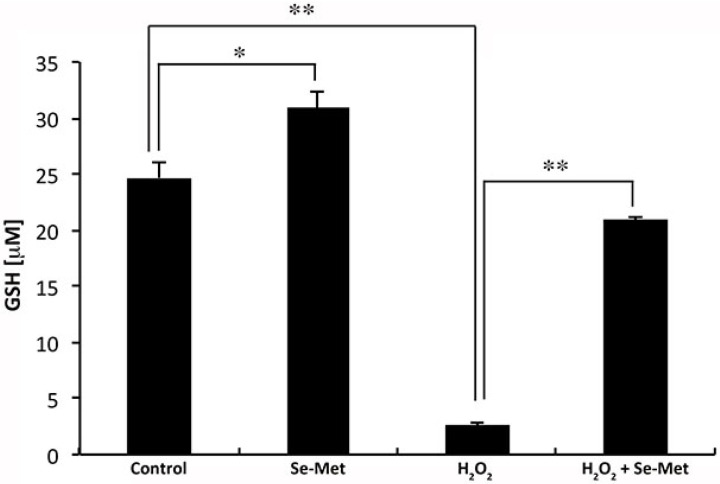
Se-Met raises intracellular levels of glutathione (GSH) protecting ARPE-19 cells against H_2_O_2_-induced GSH depletion. Oxidative stress was induced in ARPE-19 cells using hydrogen peroxide (H_2_O_2_, 500 μM) in the presence or absence of Se-Met (1 mM). Intracellular levels of glutathione (GSH) were then measured using the GSH-Glo Assay kit (Promega). (* *p* < 0.01; ** *p* < 0.001).

We have demonstrated the ability of Se-Met to induce Nrf2 and, as a result, increase the activity of the cystine transport system xc- in cultured human retinal pigment epithelial cells. System xc- is critical for cellular delivery of cysteine in the form of cystine and, hence, for the synthesis of glutathione. Indeed, others have shown that increasing system (xc-)-mediated cystine transport can result in increased intracellular GSH [10,34]. Therefore, we hypothesized the same to be true with respect to the Se-Met-induced increase in system xc- transport activity observed in our experimental system. We tested the validity of this prediction by culturing ARPE-19 cells in the presence or absence of Se-Met (1 mM) and in the presence or absence of H_2_O_2_ (500 μM). GSH concentrations were then measured. Cellular concentrations of GSH increased significantly in cells exposed to Se-Met under normal conditions (Figure 7). The effect was even more pronounced when the cells were subjected to oxidative stress. As expected, based upon our previous studies [21], levels of GSH were reduced significantly when the cells were exposed to H_2_O_2_-induced oxidative stress. This is because GSH is consumed in the process of H_2_O_2_ detoxification. However, the H_2_O_2_-induced depletion of GSH was attenuated significantly (approximately 85% inhibition) in the presence of Se-Met. These results suggest that Se-Met-induced augmentation of system xc- expression significantly enhances the ability of RPE cells to synthesize GSH and consequently to counter oxidative stress.

### 3.6. Se-Met Induces System xc- Activity also in Primary Cultures of Mouse RPE

**Figure 8:**
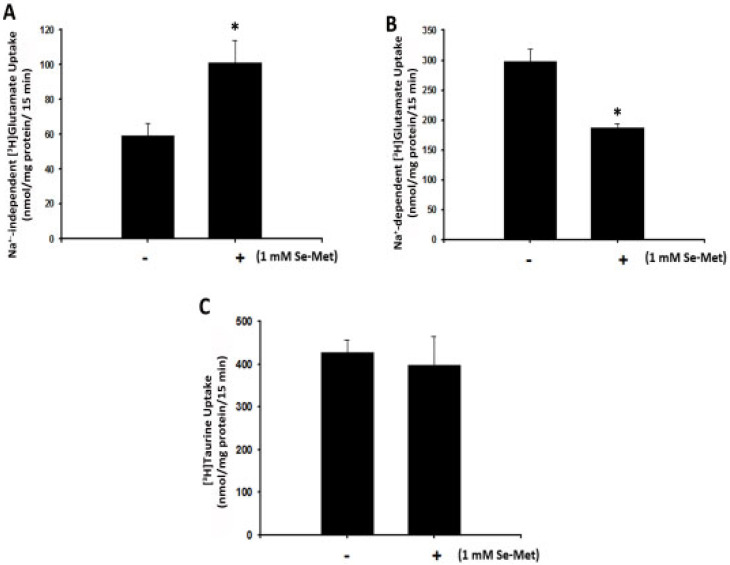
Induction of xc- by selenomethionine (Se-Met) in mouse primary RPE cells: Primary mouse RPE cells were cultured in the presence or absence of selenomethionine (Se-Met, 1 mM) for 16 h followed by measurement of [3H]-glutamate (2.5 μM) or [3H]-taurine (25 nM) uptake (15 min, 37 °C) in the presence and absence of Na+. (**A**) Na+-independent, (xc-)-specific glutamate uptake; (**B**) Na+-dependent, excitatory amino acid transporter (EAAT)-specific glutamate uptake; and (**C**) Na+-dependent taurine (TAUT) uptake. (* *p* < 0.01)**.**

The studies described thus far were carried out with ARPE-19 cells, a “transformed” RPE cell line. To confirm that the effect of Se-Met on system xc- activity in ARPE-19 cells is not an artifact of a transformed cell line, we used primary cultures of mouse RPE cells. We isolated RPE cells from normal mouse eyes, cultured them in the presence or absence of Se-Met (identically as described for ARPE-19 cells), and monitored the uptake of [3H]-glutamate in the presence and absence of Na+. Indeed, the stimulatory effects of Se-Met on Na+-independent glutamate uptake indicative of system xc- activity were observed also in these cells (Figure 8A). Also as observed with ARPE-19 cells, the inductive effect of Se-Met was specific to system xc-; EAAT activity was not increased in primary RPE cells when exposed to Se-Met. In fact, the EAAT activity, measured as Na+-dependent glutamate uptake, was reduced in cells exposed to Se-Met (Figure 8B). The specificity of the effect of Se-Met on system xc- was addressed by examining the activity of an unrelated transporter, namely the transport system responsible for the uptake of taurine. Taurine, with its robust antioxidative and other protective properties, is crucial to normal retinal health and visual function [35,36]. We found that the uptake of taurine was not altered in primary RPE cells when treated with Se-Met (Figure 8C).

## 4. Discussion

RPE cells are vital to normal retinal health and visual function, performing such critical functions by maintaining outer blood–retinal barrier properties, by nourishing and supporting photoreceptor neurons, and by regulating inflammation/immunity. As such, protecting these cells from damage is important. RPE has a formidable antioxidant defense system, of which GSH is a major component. The critical importance of GSH in retina/RPE is supported by its particularly high concentration in this tissue/cell type under normal physiologic conditions [37,38] and further evidenced by consequences associated with its decline in aging and in AMD [39,40]. GSH synthesis is largely dependent upon the availability of cysteine, a factor critically dependent, in turn, upon the activity of system xc-. Therefore, strategies to enhance the expression/activity of this transport system may be of benefit in the protection of retinal cells against oxidant-induced injury. Here, we show Se-Met, an organic form of selenium, to be very effective in this regard. Specifically, we demonstrate the induction of Nrf2, a critical regulator of the cytoprotective response during oxidative stress, in association with exposure of cultured human RPE cells to Se-Met. Though we did not directly evaluate the effect of Se-Met on the interaction between Nrf2 and Keap 1, it is well documented that Nrf2 resides in the cytoplasm in association with Keap 1 when there is no oxidative stress, but the complex dissociates during oxidative stress to release Nrf2, which then translocates into the nucleus to induce its target genes [41,42]. Interestingly, Se-Met may not only facilitate the dissociation of Nrf2 from Keap 1 but also enhance its expression. Our studies show that the total cellular levels of Nrf2 are increased upon exposure of the cells to Se-Met. If the effect of Se-Met involves only dissociation of the Nrf2/Keap 1 complex, the total levels of Nrf2 are expected to remain the same. SLC7A11, the gene coding for xCT, the functional subunit of the xc- transporter, is a direct target for Nrf2 as evidenced from SLC7A11-specific promoter-reporter assays. PCR and northern blot studies strongly support the conclusion that the activity of system xc- is increased by Se-Met at least partly via enhanced transcription of SLC7A11. This is supported by the increase in density of the transporter protein in the plasma membrane. This however does not appear to be the only mode of action of Se-Met. The observed increase in substrate affinity in Se-Met-treated cells is intriguing. The increase in maximal velocity can be explained on the basis of the increased transporter density in the plasma membrane, but the change in substrate affinity was unexpected. This could only be due to some unknown changes in the transporter protein itself. One possibility is that the transporter activity is modulated by posttranslational changes such as thiol/disulfide interchange. Since the cellular levels of GSH are altered by Se-Met, it is possible that the xCT protein is subjected to thiol/disulfide interchange, which might influence substrate affinity. Additional studies are needed to confirm this speculation.

The antioxidant properties of selenium-containing compounds like Se-Met are thought to be due primarily to the role of selenium as a constitutive metal cofactor in selenoproteins like the GPXs and TXNRDs. In addition, Se-Met induces the expression of GPX1 and TXNRD1 in ARPE-19 cells (Figure 1D). Our present findings unravel another interesting mechanism for the antioxidant property of Se-Met: it upregulates Nrf2, an important transcription factor that is associated with cellular antioxidant machinery. We also evaluated the consequences of Se-Met-induced upregulation of the transport system xc- in terms of cellular glutathione status. Even under conditions not associated with oxidative stress, Se-Met increases the cellular levels of GSH, but the effect is much greater under conditions of oxidative stress. We do not know whether the former property, the Se-Met-induced upregulation of selenoprotein expression, is related to or directly impacts the latter, the Se-Met-induced stimulation of system xc- activity; however, in sum, our findings are highly supportive of the robust antioxidant properties of Se-Met in RPE and the potential usefulness of compounds of this nature in retina.

Though at present antioxidant therapy is the standard recommendation for patients with dry AMD, there are conflicting opinions with regard to the true benefit, or lack thereof, of such therapy in terms of slowing/preventing progression of AMD. Furthermore, among those supportive of exogenous antioxidant vitamin/mineral supplementation, confusion exists as to what should be the appropriate makeup of the cocktail. At present, there is no selenium, in any form, present in Age-related Eye Disease Study (AREDS I and AREDS II) formulations [43], and to our present knowledge, there is no plan to incorporate selenium as such [44]. This is not because of any concrete evidence of a lack of gain in terms of antioxidant benefit or the presence of unwanted detrimental effects but rather is likely due in part to the large number of inconsistencies across the various studies conducted to date [40,45,46]. There are a large number of reports, clinical and experimental, touting the beneficial effects of selenium therapy in other diseases in which oxidative stress and resultant cellular damage are majorly involved (e.g., various types of cancer, HIV, and Alzheimer’s, etc.) [47,48,49]. Mechanisms provided to explain the beneficial effects of selenium in these paradigms include induction of Nrf2/phase II antioxidant signaling, regulation of p53-ERK1/2 activation, and attenuation of amyloid beta production and resultant cell death [50,51,52,53]. Many of these processes are congruent with our present findings in RPE and would be of substantial benefit in AMD to protect against oxidant-induced injury to RPE. Indeed, the critical biologic importance of selenium is without question, evidenced not only by the positive or beneficial effects associated with its supplemental use but also by the clinical consequences associated with its deficiency. Alternately, however, too much selenium can be toxic [54]. Selenium is available in a variety of different formulations; however, not all seleno-compounds are “created equal” with respect not only to their ability to raise serum selenium concentrations but also to their antioxidant and toxicological properties. This is supported by our present demonstration of the differential effects of Se-Met and Se-Cys on system xc- activity in RPE; Se-Met is much more potent than Se-Cys in stimulating xc- activity. Additionally, in comparison to other seleno-compounds, many of which have been shown to be cytotoxic at micromolar concentrations, clinical and experimental studies indicate that Se-Met can be used effectively at high doses with little associated toxicity [53,55,56]. In fact, in the present study, we exposed cultured human RPE cells to Se-Met at concentrations ranging from 100 μM up to 2.5 mM; significant stimulatory effects on system xc- were observed at all concentrations tested, and cell viability was not significantly affected even at higher doses (Figure 3C). Collectively, these factors increase the attractiveness of Se-Met as a potential therapeutic drug for use in retina. Studies by others provide added support for the use of organic selenium for this very purpose [57,58,59,60]. However, additional studies focusing on the in vivo effects of Se-Met in retina/RPE and the optimal dosages for such are required before this compound can be included either alone or in combination with other antioxidant therapies in human patients.

## 5. Conclusions

The current study demonstrates that selenomethionine, the major form of dietary selenium in humans, protects RPE cells from pro-oxidant injury via a multimodal Nrf2-related mechanism. Oxidative stress is a major component of pathology development in retina consequent to aging and degenerative diseases such as age-related macular degeneration and diabetic retinopathy. RPE is prominently impacted in these conditions. Thus, future studies to validate the collective benefit of this compound in preclinical models of aging, RPE, and outer retinal dysfunction and/or degeneration are highly warranted given the potential broad utility of selenomethionine therapy.

## Data Availability

All datasets generated for this study are included in the article/Supplementary Materials.

## Supplementary Materials

The following are available online at https://www.mdpi.com/2076-3921/10/1/9/s1, Table S1: Primer sequences used for RT-PCR assays: human GCLC-NM_001197115.2, human GCLM-NM_001308253.2, human HO-1-NM_002133.3, human GSTA1-NM_145740.5; human GSTA2-NM_000846.5, human GPX1-NM_000581.3, human TXNRD1-NM_182742.3, and human NQO1-NM_000903.3.
