# Supplementary material for: Selenomethionine (Se-Met) Induces the Cystine/Glutamate Exchanger SLC7A11 in Cultured Human Retinal Pigment Epithelial (RPE) Cells: Implications for Antioxidant Therapy in Aging Retina
Source: Antioxidants (Basel). 2020 Dec 24;10(1):9. doi: 10.3390/antiox10010009 (PMC7823377; doi:10.3390/antiox10010009)
Supplement: Supplementary file 1 [file antioxidants-10-00009-s001.pdf]

Table S1. Primer sequences used for RT-PCR assays.

Gene	Primer Sequence	Expected Product Size (bp)
Human GCLC	**FWD:** 5′-AAC CCA AAC CAT CCT ACC CT -3′<br>**REV:** 5′-TCA TCC ATC TGG CAA CTG TC -3′	1363
Human GCLM	**FWD:** 5′- TAT CAG TGG GCA CAG GTA AA -3′<br>**REV:** 5′-CAC AAT GAC CGA ATA CCG -3′	237
Human HO-1	**FWD:** 5′-ATT GCC AGT GCC ACC AAG TTC AAG -3′<br>**REV:** 5′-ACG CAG TCT TGG CCT CTT CTA TCA -3′	106
Human GSTA1	**FWD:** 5′- GAT GTT CCA GCA AGT GCC AAT GGT -3′<br>**REV:** 5′- ACG GGC AGA AGG AGG ATC ATT TCA -3′	183
Human GSTA2	**FWD:** 5′- ACG GAC AAG ACT ACC TTG TTG GCA -3′<br>**REV:** 5′- TGT GGG CAG GTT ACT GAT TCT GGT -3′	152
Human GPX1	**FWD:** 5′-AGC AGA ACG CCA AGA ACA AA -3′<br>**REV:** 5′-TTC CGA CAC ACC GGA GAC – 3′	223
Human TXNRD1	**FWD:** 5′- TTT CTC CTT GCC TTA CTG CC -3′<br>**REV:** 5′- AAG CCCACA ACA CGT TCA -3′	765
Human NQO1	**FWD:** 5′- TGC TTA CAC TTA CGC TGC C -3′<br>**REV:** 5′- TGT GCC CAA TGC TAT ATG TC -3′	212

glutamate-cysteine ligase catalytic subunit, GCLC; glutamate-cysteine ligase regulatory subunit, GCLM; heme-oxygenase 1, HO-1; glutathione S-transferase A1, GSTA1; glutathione S-transferase A2, GSTA2; thioredoxin reductase 1, glutathione peroxidase 1, GPX1; TXNRD1; NAD(P)H dehydrogenase quinone 1, NQO1
